# Physiological Responses to Thermal Stress in the Liver of *Gymnocypris eckloni* Revealed by Multi-Omics

**DOI:** 10.3390/ani15223272

**Published:** 2025-11-12

**Authors:** Miaomiao Nie, Weilin Ni, Zhenji Wang, Dan Liu, Qiang Gao, Cunfang Zhang, Delin Qi

**Affiliations:** 1 State Key Laboratory of Plateau Ecology and Agriculture, Qinghai University, Xining 810016, China; 2 Fishery Environmental Monitoring Station of Qinghai Province, Xining 810012, China

**Keywords:** thermal stress, *Gymnocypris eckloni*, multi-omics, climate change

## Abstract

Climate-change-induced thermal stress poses a significant threat to cold-adapted aquatic species, particularly fish endemic to high-altitude ecosystems such as *Gymnocypris eckloni*, which is native to the Qinghai-Tibetan Plateau. To elucidate the molecular and metabolic mechanisms underlying their response to elevated temperatures, we integrated RNA-seq, miRNA-seq, and LC-MS-based metabolomic analyses of liver tissue from fish exposed to chronic thermal stress (HT) versus control (CT) conditions. Although no significant differences were observed in growth parameters, histopathological examination revealed structural damage under heat stress. Transcriptomic analysis identified widespread dysregulation of genes involved in energy metabolism, with significant downregulation of pathways related to amino acid, fatty acid, glucose, and oxidative phosphorylation. In contrast, upregulated DEGs were enriched in N-glycan biosynthesis, protein processing in the endoplasmic reticulum, and phagosome. Concomitant miRNA profiling revealed differentially expressed miRNAs, including miR-196a-5p, miR-132-3p, and miR-181b-5p, which were predicted to regulate key metabolic genes such as *ugt1a1*, *pepck*, and *calr*. Metabolomic analysis further demonstrated significant alterations in metabolic profiles, with glutathione metabolism, tryptophan metabolism, steroid hormone biosynthesis, and pyruvate metabolism emerging as central pathways in the heat stress response. Integrated multi-omics analysis confirmed coordinated regulation of these pathways, highlighting the critical role of glutathione and tryptophan, as well as disruptions in purine and energy metabolism. The DEMiR-DEG-DEM networks involving miR-196a-5p-*pepck*-PEP, miR-133a-3p-*gne*-UDP-GlcNAc, and miR-132-3p-*ugt1a1*-Bilirubin may play an important role in thermal stress. This study provided a new perspective on the molecular, regulatory, and metabolic adaptations of *Gymnocypris eckloni* to thermal stress, identifying potential biomarkers and regulatory networks that may inform conservation strategies for cold-water fish under global warming.

## Simple Summary

As global temperatures rise, cold-adapted species such as *Gymnocypris eckloni*, endemic to the Tibetan Plateau, are increasingly threatened by thermal stress. This study investigates the physiological and molecular responses of *Gymnocypris eckloni* to prolonged heat exposure, with a focus on liver adaptation mechanisms. While no significant changes in growth were observed, histological and multi-omics analyses revealed substantial cellular and metabolic remodeling in the liver. The fish exhibited a shift in energy allocation, suppressing anabolic processes while enhancing stress-responsive pathways involved in protein homeostasis and cellular protection. Regulatory mechanisms, including microRNA-mediated gene expression, appear to play a key role in fine-tuning these adaptive responses. Overall, *Gymnocypris eckloni* demonstrates a complex but finite capacity to maintain physiological stability under elevated temperatures. These findings enhance our understanding of how climate change affects cold-adapted species and could guide future conservation efforts.

## 1. Introduction

As ectotherms, fish are particularly susceptible to water temperature, which plays a central role in their survival by governing vital processes like growth, development, metabolism, and immunity [1,2,3,4]. Fish exhibit an optimal thermal range within which they can adequately adapt [5,6,7]; however, when temperatures exceed their tolerance limits, their growth and even survival may be severely compromised, especially under high water temperature, such as brown trout *Salmo trutta* [8] and Pacific salmon [9]. Climate change is projected to place nearly half of all freshwater fish species at risk of endangerment or extinction [10,11]. Rising global temperatures are altering the thermal regimes of riverine ecosystems, posing a significant threat to freshwater biodiversity [12]. Fish species are affected both directly and indirectly by the changing thermal conditions of water [13,14].

Transcriptomic analysis has revealed key regulatory genes involved in thermal stress response in various fish species, such as *Megalobrama amblycephala* [15], rainbow trout [16], *Haliotis discus hannai* [17], *Lateolabrax maculatus* [18], and *Epinephelus fuscoguttatus* [19]. Metabolomic studies in *Brachymystax lenok* indicate that alterations in energy metabolism, as well as in the levels of L-carnitine, acylcarnitine esters, glycerophospholipids, and glutamate, are associated with thermal stress [20,21]. While in *Micropterus salmoides*, metabolomics showed that steroidal saponins can ameliorate hepatic lipid metabolism under elevated temperatures [22]. Integrated metabolomic and transcriptomic analyses, as applied in *Triplophysa siluroides*, have demonstrated that enhanced purine metabolism contributes to cellular protection during heat stress [23]. Such multi-omics approaches allow for a systems-level understanding of the gene–metabolite interactions that mediate thermal stress responses in cold-water fish.

Over the past six decades, the Tibetan Plateau has experienced the most rapid climate warming in China [24,25], leading to rising water temperatures and threatening the habitats of cold-adapted fish species. This trend poses a particularly significant challenge for Schizothoracine fishes, which are endemic to high-altitude environments and uniquely adapted to the cold conditions of the Tibetan Plateau [26,27,28]. However, research on the impacts of climate warming on these species remains limited. *Gymnocypris eckloni*, a cold-water fish within Schizothoracinae, has an optimal growth temperature of 14~16 °C [29], and lives in high-altitude waters of the upper Yellow River [26,27]. In these ecosystems, *Gymnocypris eckloni* plays a crucial ecological role and is also an important economic fish species in Qinghai Province. To date, most studies of *Gymnocypris eckloni* have focused on its phylogenetics [30,31], hypoxia tolerance [28], and adaptations to cold environments [32,33]. Interestingly, rising water temperatures due to global warming may confer certain benefits to *Gymnocypris eckloni*, such as improved spawning success and enhanced larval survival [34]. Acute heat stress (27 °C) has been shown to significantly increase swimming speed, distance, and respiration rate, accompanied by alterations in cardiac gene expression [35]. Furthermore, acute heat exposure (28 °C for 12 h) induces tissue damage in the intestine [36,37] and liver [38], and tryptophan metabolism has been implicated as a key regulator of chronic heat stress responses in this species [38], yet a comprehensive understanding of its long-term thermal acclimation mechanisms remains limited.

As a central hub of metabolism, the liver plays a pivotal role in maintaining energy homeostasis under stress through the coordinated regulation of carbohydrate, lipid, and protein metabolic pathways [39,40]. Previous studies have demonstrated that both acute and chronic heat stress provoke significant structural and metabolic alterations in the liver [38,41]. In the present study, we exposed *Gymnocypris eckloni* to chronic heat stress and integrated miRNA, mRNA, and metabolite profiling to investigate the liver’s response at the transcriptomic and metabolomic levels. Furthermore, we elucidated the regulatory roles of miRNA–mRNA–metabolite networks in thermal adaptation. This study provided a comprehensive framework to understand the molecular mechanisms underlying thermal tolerance in cold-adapted fish species.

## 2. Materials and Methods

### 2.1. Fish and Heat Acclimation

Healthy specimens of *Gymnocypris eckloni* (2.5 years old, total length:16.98 ± 1.38 cm) were obtained from the Fisheries Promotion Center of Qinghai Province. The fish were transported to our laboratory in aerated plastic containers and acclimated in flow-through aquaria for two weeks at 14~16 °C. They were cultivated in air-pumped water (day/night: 14 L/10 D, dissolved oxygen: approximately 5 mg/L). The fish were fed a commercial diet twice daily to satiation. All procedures involving animals followed standard ethical guidelines. The study protocol was specifically reviewed and approved by the Animal Care and Use Committee of Qinghai University, China (SL-2023027).

**Figure 1:**
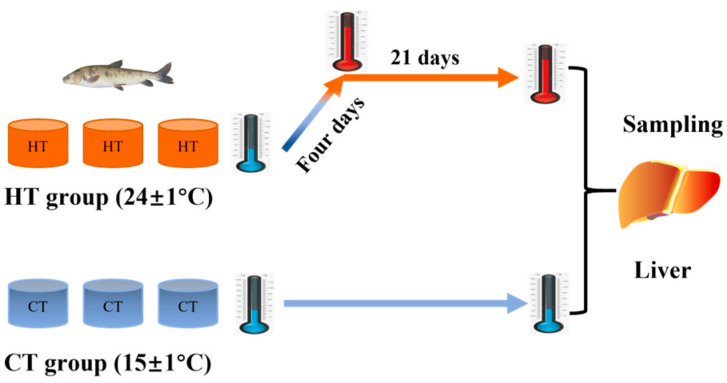
The schematic diagram of thermal stress of *Gymnocypris eckloni*.

Sixty individuals were randomly assigned to two experimental groups: a control temperature (CT) group (15 ± 1 °C) and a high-temperature (HT) group (24 ± 1 °C). Each group was held in three replicate tanks (0.2 m^3^) per group, with ten fish each. The temperature in the HT group was gradually increased from 15 °C to 24 °C over four days (at a rate of approximately 2.25 °C per day) and then maintained at 24 °C for 21 days (Figure 1). Based on the water temperature of fish breeding ponds in Qinghai Province during summer, the temperature for the HT group was set at 24 ± 1 °C. There was no death of fish during the experiment. Prior to tissue collection, body weight (BW), total length (TL), and body length (BL) of ten fish were measured. Then six fish per group were euthanized with MS-222 (Sigma-Aldrich, Wuxi, China), and livers were excised on ice. For each group, six liver samples were immediately snap-frozen in liquid nitrogen and stored at −80 °C for subsequent LC-MS-based metabolomic analysis. Among these, three samples per group were used for RNA-seq and miRNA-seq analysis. The liver tissues were also used for histological analysis. The condition factor (CF), a proxy for fish health and overall metabolic status, was calculated as 100× (BW × BL^−3^), based on the mean values of each group.

### 2.2. Histological Analysis

Liver tissues were also collected from *Gymnocypris eckloni* in summer from natural breeding ponds at the Fisheries Promotion Center of Qinghai Province. Tissues were fixed in Davidson’s fixative, dehydrated through a graded ethanol series, and embedded in paraffin. After being cut into a series of sagittal and cross sections (5 μm), they were stained with hematoxylin and eosin (H&E) and observed under a Leica microscope (Wetzlar, Germany).

### 2.3. mRNA and Small RNA Sequencing

Total RNA was extracted from liver tissue using TRIzol^®^ Reagent (Invitrogen, Carlsbad, CA, USA) according to the manufacturer’s instructions. RNA concentration and purity were assessed using a NanoDrop ND-1000 spectrophotometer (Thermo Fisher Scientific, Wilmington, DE, USA), with samples exhibiting A260/A280 ratios between 1.8 and 2.1 considered acceptable. RNA integrity was also evaluated before being used for downstream applications. Only high-quality RNA samples were used for constructing both mRNA sequencing (RNA-seq) and small RNA sequencing (small RNA-seq) libraries.

Sequencing libraries for RNA-seq and small RNA-seq were constructed as described in our previous study [32]. Raw data (raw reads) of fastq format were first processed through in-house perl scripts. Clean data were obtained after quality control of raw data. Then the raw reads were deposited in the NCBI Sequence Read Archive (SRA) (SRR19178216, SRR19178217, SRR19178218, SRR19178219, SRR19178220, SRR19178221, SRR19177530, SRR19177531, SRR19177532, SRR19177533, SRR19177534, and SRR19177535). RNA-seq clean reads and the small RNA tags were mapped to reference sequence the *Gymnocypris eckloni* reference genome [33] using HISAT2 (v2.0.5) and Bowtie (bowtie-0.12.9) software, respectively.

### 2.4. Identification and Functional Enrichment Analysis of Differentially Expressed Genes, miRNAs, and miRNA Targets

Differentially expressed genes (DEGs), differentially expressed miRNAs (DEMiRs), and their target genes were identified from RNA-seq and small RNA-seq data using the DESeq2 package (v1.20.0 for mRNAs; v3.0.3 for miRNAs). DEGs were defined by an adjusted *p*-value < 0.05 and |log_2_(fold change)| > 0, while DEMiRs were identified using a significance threshold of *p*adj < 0.05. Gene Ontology (GO) and Kyoto Encyclopedia of Genes and Genomes (KEGG) pathway enrichment analyses were performed to characterize the functional and biochemical roles of the DEGs and predicted target genes of DEMiRs. GO enrichment was conducted using the clusterProfiler R package (v3.4.4) and GOseq (v1.12). KEGG analysis was performed using clusterProfiler and KOBAS (v2.0) to assess the statistical overrepresentation of DEGs and miRNA targets in known biological pathways.

### 2.5. Quantification of Genes and Statistical Analysis

The expression levels of selected differentially expressed mRNAs and miRNA target genes were validated by real-time quantitative RT-PCR (qPCR) using RNA isolated from liver samples. As a cellular energy sensor, AMPK responds to low ATP levels. Upon activation, it positively regulates signaling pathways that restore cellular ATP supply, such as fatty acid oxidation and autophagy. Conversely, AMPK negatively regulates ATP-consuming biosynthetic processes, including gluconeogenesis, lipid synthesis, and protein synthesis [42]. So, we also detected the expression levels of AMPK subunits (*ampkα1*, *ampkα2*, *ampkβ1*, *ampkβ2*, *ampkγ1*, and *ampkγ2*), and key downstream target genes, including *pgc1α*, *tfam*, *nrf*, and *hmgcr*, were analyzed in liver tissues of *Gymnocypris eckloni* under thermal stress. Gene-specific primers (Table S1) were designed based on sequencing data. *β*-*actin* was selected as an internal reference gene. qPCR was carried out in a LightCycler 480 instrument with SYBR Green PCR Master Mix (Takara, Kusatsu, Japan), under the following thermal cycling conditions: initial denaturation at 95 °C for 15 s, followed by 35 cycles of 95 °C for 5 s, annealing at 60 °C for 15 s, and extension at 72 °C for 25 s.

Data are presented as means ± standard error. Statistical significance of differential expression between HT and CT groups was determined by a two-tailed *t* test, with a *p*-value < 0.05 deemed statistically significant.

### 2.6. UHPLC-MS/MS Analysis, Metabolite Identification, and Data Analysis

Metabolites extraction from liver tissue of *Gymnocypris eckloni* was performed according to the method described in the reference [43]. The metabolites in the supernatant were injected into the LC-MS/MS system for analysis. The UHPLC-MS conditions, including column type, gradient program, ionization parameters, and mass acquisition settings, were adopted from previously validated metabolomics protocols [41,44], with minor adjustments optimized for the liver metabolome of *Gymnocypris eckloni*.

Raw UHPLC-MS/MS data were processed using Compound Discoverer 3.1 (ThermoFisher) for peak detection, alignment, and quantification. Metabolite molecular formulas were predicted based on additive ions, molecular ion peaks, and fragment ions. Putative identifications were achieved by matching against the mzCloud, mzVault, and MassList databases. Normalized data were analyzed using R (v3.4.3), Python (v2.7.6), and CentOS (6.6); non-normally distributed data were normalized using area normalization.

Metabolites were annotated via the KEGG, HMDB, and LIPIDMAPS databases. Principal component analysis (PCA) and partial least squares discriminant analysis (PLS-DA) were performed using metaX (v1.4.2). Differential metabolites were defined by VIP > 1, *p* < 0.05 (univariate *t*-test), and fold change ≥ 2 or ≤0.5. Volcano plots (log_2_FC vs. −log_10_P) were used to visualize significant metabolites. Functional and pathway enrichment analyses were conducted using KEGG databases.

### 2.7. Interaction Analysis of mRNA, miRNA, and Metabolite

An integrated miRNA–mRNA regulatory network analysis was constructed by combining DEGs identified from RNA-seq with differentially expressed miRNAs from small RNA-seq, along with their predicted target genes. Concurrently, DEGs and differential metabolites were jointly mapped to the KEGG pathway database to identify significantly enriched shared pathways. This integrative approach enabled the identification of key biochemical and signaling pathways co-regulated by transcriptomic and metabolomic changes, thereby facilitating a deeper exploration of the functional interactions between the two omics datasets.

### 2.8. Statistical Analysis and Pathway Analysis

The positive and negative data were combined to obtain a combined data which was imported into SIMCA software package (version 14.0, Umetrics, Umeå, Sweden) for principle component analysis (PCA) and orthogonal partial least squares discriminant analysis (OPLS-DA). The Hotelling’s T2 region, shown as an ellipse in score plots of the models, defined the 95% confidence interval of modeled variation. Afterwards, a 7-round cross-validation was performed. One seventh of the samples were excluded from the model in each round so as to validate the model. On the basis of variable importance in projection (VIP) values > 1.0 obtained from the OPLS-DA model and *p*-values < 0.05 from a two-tailed Student’s *t*-test, differential metabolites were identified. The KEGG database was applied to link these metabolites to metabolic pathways.

## 3. Results

### 3.1. Fish Growth Parameters and Histopathological Change

**Figure 2:**
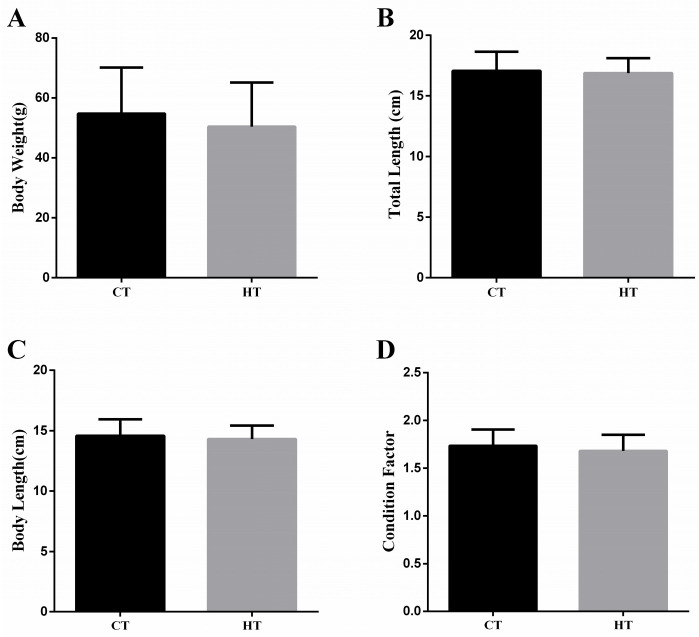
Effect of heat stress on body weight (**A**), total lengths (**B**), body length (**C**), and condition factor (**D**). Data shown are means ± SEM, *n* = 10.

**Figure 3:**
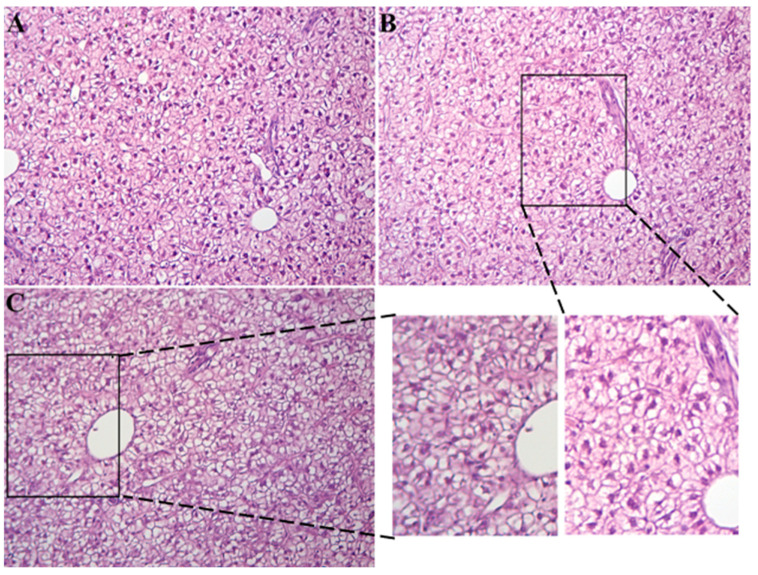
The liver structure of *Gymnocypris eckloni* in different treatments. (**A**–**C**) represent CT group (**A**), HT group (**B**), and fish liver in summer (**C**).

Following treatment, BW, TL, and BL of fish were recorded, and CF was calculated. We found fish individuals in HT had lower BW, TL, BL, and CF than that in CT (*p* > 0.05) (Figure 2). Histopathological changes in the liver of *Gymnocypris eckloni* under heat stress are shown in Figure 3. In the CT group (Figure 3A), hepatocytes exhibited well-defined cell boundaries, with large, round, centrally located nuclei and a clearly organized cytoarchitecture, consistent with normal hepatic morphology. In contrast, livers from the HT group (Figure 3B) displayed mild histopathological alterations, including displacement of some nuclei from their typical central position. In fish sampled during summer (Figure 3C), the liver tissues showed vacuolar degeneration, a fuzzy cell shape, and nuclear pyknosis.

### 3.2. RNA-Seq, Small RNA-Seq, and Analysis

RNA-seq and small RNA-seq libraries were prepared from each specimen in both the CT and HT groups. For each group, approximately 45 million (M) raw RNA-seq reads were generated, yielding ~43 million clean reads after quality filtering (Table S2). Of the clean reads, over 75% were successfully mapped to the *Gymnocypris eckloni* reference genome (Table S2). Small RNAs ranging from 18 to 35 nucleotides (nt) were sequenced, resulting in approximately 11 million and 13 million clean small RNA reads for the CT and HT groups, respectively (Table S3). In total, we identified 529 miRNAs, comprising 289 known and 240 novel mature miRNAs.

**Figure 4:**
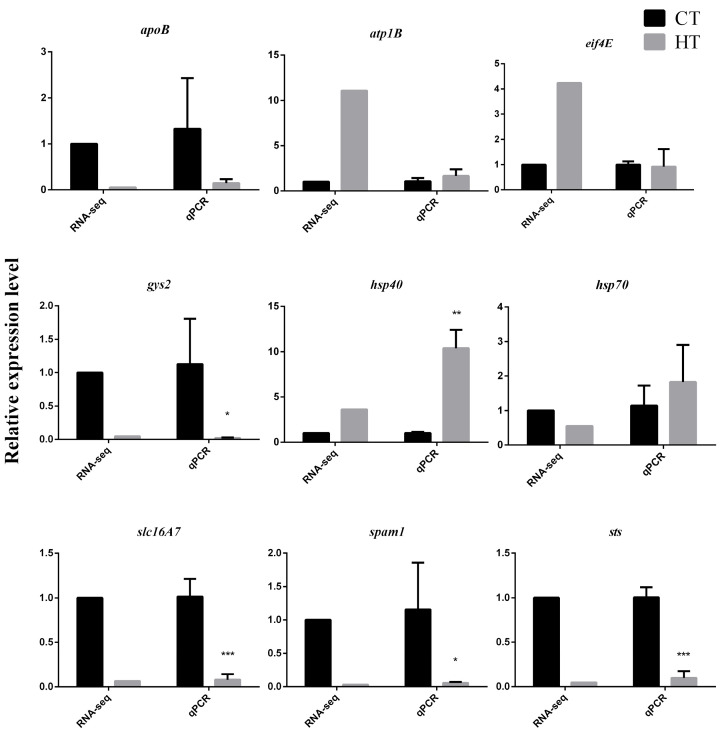
Validation of RNA-seq results by qPCR. * *p* < 0.05; ** *p* < 0.01; *** *p* < 0.001.

Following heat acclimation, we obtained 1576 upregulated and 1685 downregulated genes in the HT group response to thermal stress (Figure S1A). Compared to the CT group, small RNA sequencing revealed 23 upregulated and 14 downregulated miRNAs in the HT group (Figure S1B). To validate the RNA-seq results, the expression levels of selected genes, including *atp1b*, *hsp40*, *hsp70*, etc., were assessed by quantitative real-time PCR. The qPCR results showed a high degree of correlation with the RNA-seq data, confirming the reliability and accuracy of the transcriptomic profiling (Figure 4).

#### Pathways Involving DEGs and miRNA-Targets

**Figure 5:**
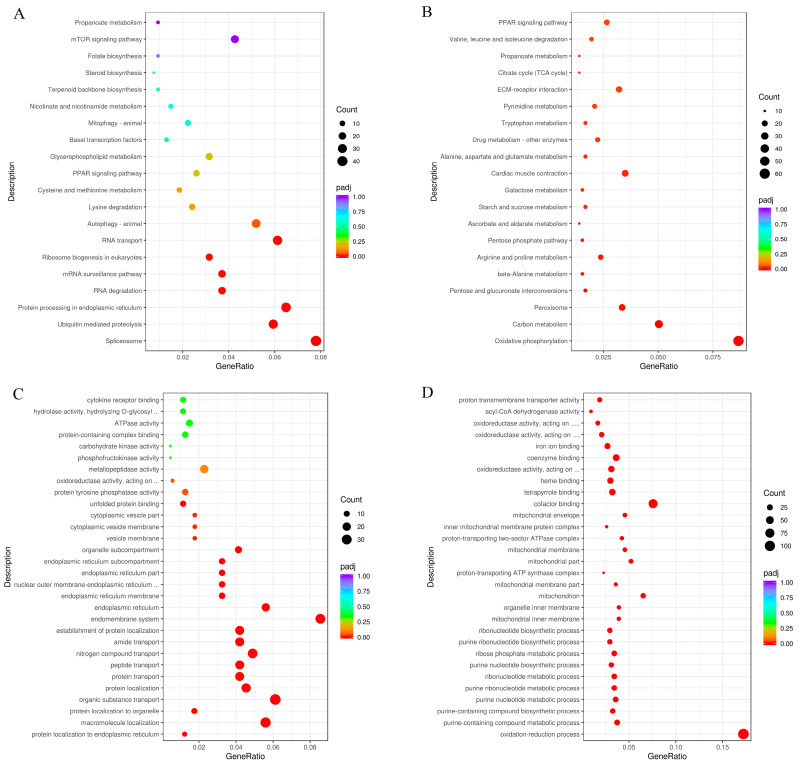
KEGG pathway (**A**,**B**) and GO (**C**,**D**) classifications for upregulated (**A**,**C**) and downregulated (**B**,**D**) DEGs, respectively.

KEGG pathway analysis showed that upregulated DEGs were significantly enriched in pathways including protein processing in the ER, protein export, phagosome, N-Glycan biosynthesis, herpes simplex virus 1 infection, and various types of N-glycan biosynthesis pathways (*p*adj < 0.05) (Figure 5A). In addition, most downregulated DEGs were identified in amino acid metabolism (e.g., valine, leucine and isoleucine degradation, glycine, serine and threonine metabolism, tryptophan metabolism, beta-alanine metabolism, lysine degradation, tyrosine metabolism, etc.), fatty acid metabolism (e.g., fatty acid degradation, fatty acid metabolism, PPAR signaling pathway, biosynthesis of unsaturated fatty acids, steroid hormone biosynthesis, fatty acid elongation, glycerolipid metabolism, and glycerophospholipid metabolism), glucose metabolism (e.g., glycolysis/gluconeogenesis and fructose and mannose metabolism), and energy metabolism (e.g., pyruvate metabolism, TCA cycle, and oxidative phosphorylation). Moreover, vitamin (e.g., retinol and vitamin B6) metabolism and primary bile acid biosynthesis also warranted attention (Figure 5B).

GO analysis showed that upregulated DEGs were primarily enriched in protein localization to endoplasmic reticulum, organic substance transport, endomembrane system, endoplasmic reticulum membrane, oxidoreductase activity, unfolded protein binding, and protein tyrosine phosphatase activity. In addition, GO enrichment analysis revealed that downregulated DEGs were predominantly associated with biological processes such as the oxidation-reduction process, purine-containing compound biosynthetic process, and purine nucleotide metabolic process; cellular components including the mitochondrial inner membrane and mitochondrial membrane part; and molecular functions related to cofactor binding (Figure 5C,D). It is worth noting that long-term heat stress appears to suppress energy metabolism in *Gymnocypris eckloni* and damage the inner membrane system.

**Figure 6:**
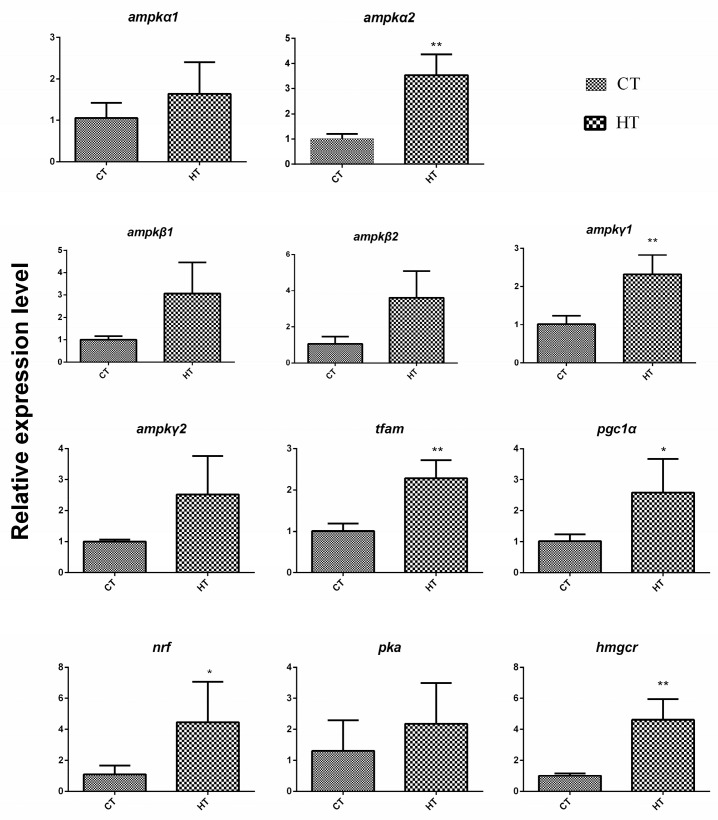
The expression levels of *ampk* and its downstream genes in liver tissues of *Gymnocypris eckloni* under thermal stress. Note, CT, control temperature group; HT, high temperature group. *: *p <* 0.05, **: *p <* 0.01. *n* = 4.

In eukaryotes, the AMPK signaling pathway plays a central role in maintaining this metabolic homeostasis. In this study, the expression levels of AMPK subunits (*ampkα1*, *ampkα2*, *ampkβ1*, *ampkβ2*, *ampkγ1*, and *ampkγ2*), and key downstream target genes, including *pgc1α*, *tfam*, *nrf*, *tfam*, and *hmgcr*, were analyzed in liver tissues of *Gymnocypris eckloni* under thermal stress. The results showed that the expression levels of all these genes were raised when the *Gymnocypris eckloni* was under thermal stress (Figure 6). This coordinated upregulation suggests a compensatory activation of the AMPK-mediated energy-sensing pathway in response to thermal fluctuations, potentially to restore cellular energy balance following metabolic disruption.

### 3.3. Integrated Analysis of DEGs and DEMiRs, and Potential miRNA-mRNA Regulatory Networks

To explore potential regulatory interactions between miRNAs and mRNAs under thermal stress, an integrated miRNA-mRNA network analysis was performed. A total of 91 DEGs overlapped with predicted targets (intersection genes) of DEMiRs in the HT group vs. the CT group. Among them, 33 were upregulated and 58 were downregulated in the HT group. Of these, 46 intersection genes (15 upregulated and 31 downregulated) were associated with upregulated DEMiRs (miR-29a, -29b, -129-5p, -196a-5p, -212, -212-5p, -216a, -217, -132-3p, -133a-3p, -133b-3p, -205-5p, novel_114, novel_160, and novel_582). Additionally, 45 intersection genes (18 upregulated and 27 downregulated) were linked to the downregulated DEMiRs (let-7b, novel_622, miR-125b-1-3p, -125b-2-3p, -301b-5p, -181a-5-3p, and miR-181b-5p) (Table S5). KEGG analysis of the intersection genes revealed involvement in pathways such as ribosome biogenesis in eukaryotes, RNA transport, phagosome, pyruvate metabolism, ascorbate and aldarate metabolism, starch and sucrose metabolism, and vitamin B6 metabolism, and so on (Table S5).

Among the identified DEMiRs and their target DEGs, miR-181b-5p and miR-125b-2-3p may be involved in Ribosome biogenesis in eukaryotes, RNA transport, phagosome, and protein processing in ER after thermal stress. Notably, miR-132-3p, miR-205-5p, miR-196a-5p, and miR-133a-3p may regulate energy metabolism via associated genes including *ugt1a1*, *rgn*, *pepck*, and *gne* (Table 1). These interactions suggest coordinated regulation of glucose, fatty acid, and protein metabolism under thermal challenge, forming a complex regulatory network that may help maintain metabolic homeostasis in *Gymnocypris eckloni* during environmental stress.

### 3.4. Metabolite Profiling in Response to Thermal Stress

**Figure 7:**
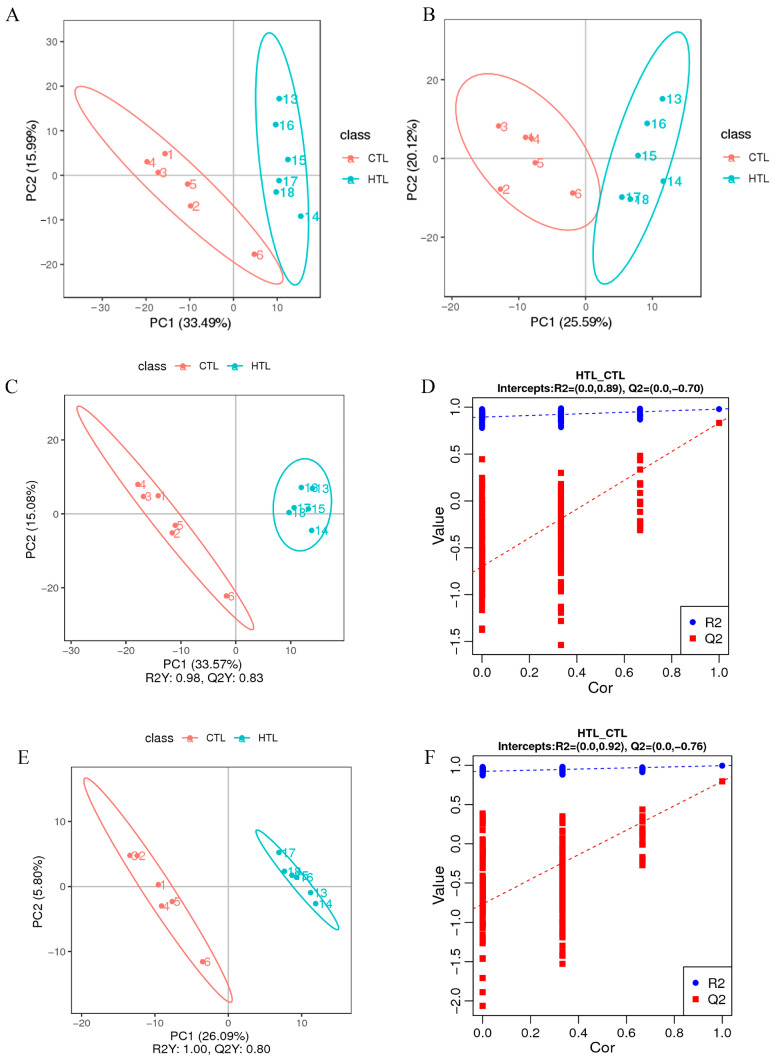
PCA and PLS-DA score plots of control and high temperature groups. Score plots from PCA (**A**,**B**) and PLS-DA (**C**,**E**) in positive and negative ion modes, along with their corresponding permutation tests (**D**,**F**).

In total, we identified 897 metabolites using LC-MS analysis. To evaluate the physiological response to heat stress, unsupervised PCA and PLS-DA were performed. The result showed obvious separations (Figure 7A,B), and differences in metabolite levels (Figure 7C–F) between the HT and the CT groups. Model validity was assessed using cumulative R^2^Y and Q^2^ values, which indicated excellent goodness of fit and high predictive accuracy for all models in both ionization modes. These results support the reliability and robustness of the PLS-DA models, confirming that high-temperature exposure induces substantial and reproducible changes in the liver metabolome of *Gymnocypris eckloni*.

#### Differential Metabolites and Metabolic Pathways in Response to Thermal Stress

**Figure 8:**
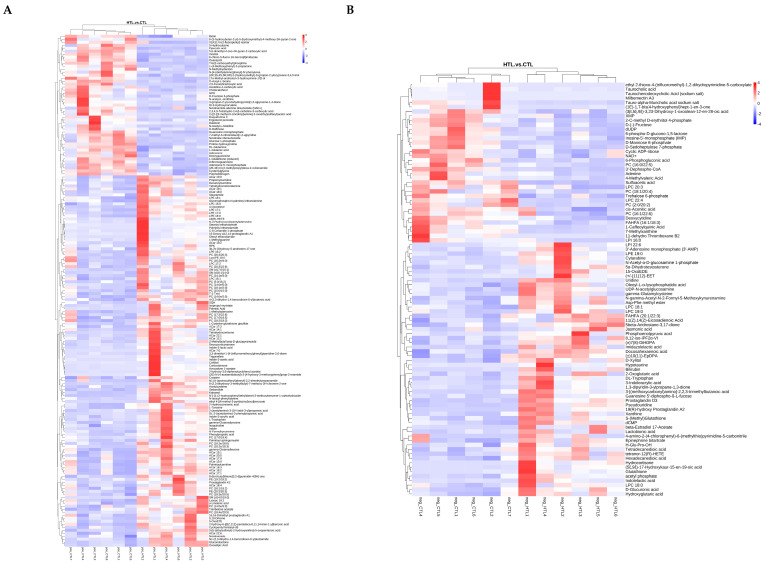
Heat map of differential metabolites in HTL following positive (**A**) and negative (**B**) modes. Color indicates the amount of metabolites from the highest (red) to the lowest (blue).

A total of 264 differential metabolites (DEMs) were identified between the HT and CT groups based on the PLS-DA model (Figure 8, Table S2), with 181 being upregulated and 83 downregulated. When stratified by ionization mode, 171 DEMs were detected in positive mode (124 up, 47 down) and 93 in negative mode (57 up, 36 down) (Table S2).

These differential metabolites were mapped to metabolic pathways using KEGG, revealing significant enrichment for the upregulated DEMs in pathways related to steroid hormone biosynthesis, tryptophan metabolism, pyruvate metabolism, aldosterone-regulated sodium reabsorption, phenylalanine, tyrosine, and tryptophan biosynthesis, among others (Table 2). For the downregulated DEMs, the relevant pathways were identified as cGMP-PKG signaling pathway, cAMP signaling pathway, AMPK signaling pathway, olfactory transduction, purine metabolism, among others (Table 2).

### 3.5. Integrated Analysis of DEMs and Transcriptome

A joint analysis of the metabolome and transcriptome confirmed that the three aforementioned metabolic pathways were significantly modulated by thermal stress (Table 2). Notably, high temperature exposure increased the liver levels of key metabolites such as phosphoenolpyruvate, creatine, and acetyl phosphate, while it decreased those of D-fructose 6-phosphate, glucose 1-phosphate, and adenosine 5′-monophosphate.

## 4. Discussion

As ectotherms, fish are particularly susceptible to water temperature, which governs their survival and distribution [1,4]. Climate change can affect fish species both directly and indirectly, especially Schizothoracine fishes, which are well-adapted to cold environments [34]. To gain deeper insights into the physiological mechanisms underlying thermal stress in *Gymnocypris eckloni*, we integrated RNA-seq, miRNA-seq, and metabolomics to characterize heat-induced metabolic alterations in the liver.

### 4.1. Growth Parameters Comparison and Histopathological Change

Fish typically reduce their feeding rate under elevated water temperatures, eventually ceasing food intake at threshold temperatures [45,46]. Consistently, *Gymnocypris eckloni* exposed to thermal stress (HT group) exhibited reduced food consumption in the present study. Fasting represents a key metabolic adaptation commonly observed across species, from yeast to mammals [41,47]. In this context, fish in the control group (CT) showed a better growth performance compared to the HT group, suggesting impaired growth performance under heat stress. We found that bile salt, taurochenodeoxycholic acid (sodium salt), hypotaurine, and taurocholic acid, which are involved in digestion and absorption, were decreased. Furthermore, the metabolism of amino acids, glucose, and fatty acids lipoic acid decreased, and primary bile acid biosynthesis was also downregulated at the transcriptional level. The liver is an essential metabolic organ [48]. Heat damages the liver tissue of fish [38,49], as was also observed in the present study, and other tissues including the heart [35] and intestine [37].

### 4.2. Transcriptional Response to Thermal Stress

To mitigate thermal damage, fish remodel various life processes to varying degrees [50]. MiRNAs are highly conserved molecules that participate positively with increased variety of biological activities through the post-transcriptional organization of gene expression, and play a role in teleost energy metabolism and environmental stress responses [51,52]. Several miRNAs have been implicated in metabolic regulation and thermal adaptation. For instance, miR-30b, miR-122, and miR-92a-3p can target key enzymes involved in glucose and lipid metabolism, contributing to cold adaptation [53]. Conversely, specific miRNAs such as bmo-miR-281-5p and novel-miR-53 are associated with heat stress responses [52,54]. In *Oreochromis niloticus*, upregulation of miR-1, miR-122, and miR-10c was observed after elevated temperatures [55], suggesting their involvement in thermal acclimation. Moreover, studies in rainbow trout have identified 20 critical miRNA-mRNA interaction pairs linked to heat stress response [56]. According to the transcriptomic data, most of the downregulated DEGs were identified in the amino acid metabolism, fatty acids metabolism, glucose metabolism, and energy metabolism, while upregulated DEGs were associated with protein processing in ER, protein export, phagosome, N-glycan biosynthesis, herpes simplex virus 1 infection, and various types of N-glycan biosynthesis pathways. These pathways were regulated by miRNA-mRNA interactions, such as miR-132-3p-*ugt1a1*, miR-196a-5p-*pepck*, and miR-125b-2-3p-*calr*.

### 4.3. Metabolic Response to Thermal Stress

KEGG pathway analysis of DEMs revealed that amino acid and lipid metabolism in the liver of *Gymnocypris eckloni* play central roles under high temperature. Amino acids serve as the building blocks of proteins and are essential for stress response [57]. In the HT group, thermal stress elevated levels of several free amino acids, including L-tryptophan, DL-tryptophan, L-tyrosine, glutathione, DL-glutamine, acetylcysteine, γ-glutamyltyrosine, and N-lactoyl-phenylalanine. This accumulation may be linked to enhanced protein processing in the ER, as suggested by transcriptomic data. Similarly, in high-altitude fish such as *Triplophysa siluroides*, increased ubiquitin-dependent proteolysis has been linked to higher concentrations of free amino acids [22], indicating a conserved metabolic strategy under environmental stress. Furthermore, creatine, a key molecule in ATP regeneration and a well-established indicator of proteolytic activity [58], was significantly increased in the present study, consistent with findings from a study on the same fish species under heat stress [38,59]. This further supports the activation of protein catabolism as an adaptive energy-supplying mechanism during heat exposure. It is worth noting that tryptophan metabolism responds to thermal stress. In the present study, tryptophan and its related metabolites, including DL-tryptophan, indole, indole-3-acetic acid, indole-3-acrylic acid, indole-3-lactic acid, indolelactic acid, and oxoadipic acid, were markedly elevated after thermal stress. Tryptophan has been shown to play an indispensable role in fish species [40,60], and recent evidence highlighted its involvement in heat stress responses in *Gymnocypris eckloni* [38].

In addition to amino acid metabolism, purine and pyrimidine metabolism were also perturbed. Purines, such as adenine and guanine, are essential components of nucleic acids and coenzymes (e.g., NADH, coenzyme A) and play vital roles in energy metabolism and signal transduction [61,62]. Adenosine 5′-triphosphate (ATP), the primary cellular energy currency, is derived from purine metabolism and fuels numerous metabolic processes. Pyrimidines, while best known as constituents of DNA and RNA, also participate in the biosynthesis of lipids, carbohydrates, and nucleotide sugars [63]. Notably, pyrimidine metabolism supports mitochondrial pyruvate oxidation, thereby facilitating lipogenesis and maintaining metabolic flexibility under stress [64].

### 4.4. Regulation Network in Response to Chronic Thermal Stress in G. eckloni

**Figure 9:**
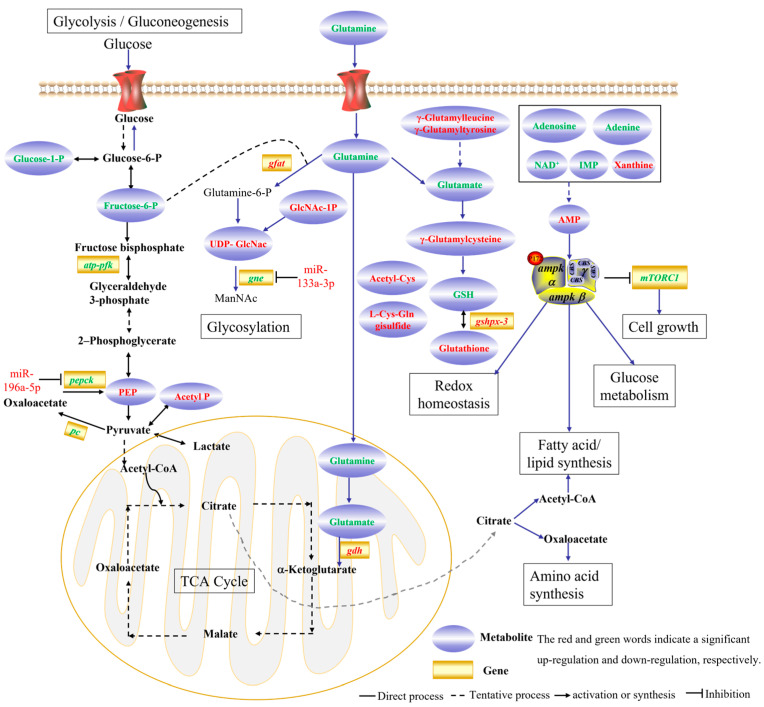
Proposed mechanisms of glutathione metabolism response to thermal stress in the *G. eckloni*.

Integrated transcriptomic, miRNA transcriptomic, and metabolomic analysis revealed that DEMiRs, DEGs, and DEMs were enriched in many overlapping or specific pathways. Among them, glutamine metabolism has attracted our attention. Glutathione metabolism played a critical role in antioxidant defense and cold stress in fish [41,47]. In the present study, glutamine and its derivatives, including gamma-glutamylcysteine, gamma-glutamylleucine, L-glutathione (reduced), and DL-glutamine, played a role in redox homeostasis, as well as in glycosylation (Figure 9). Furthermore, further studies should focus on networks such as to DEMiR-DEG-DEM networks, including miR-196a-5p-*pepck*-PEP, miR-133a-3p-*gne*-UDP-GlcNAc, and miR-132-3p-*ugt1a1*-Bilirubin, when investigating thermal stress response in fish.

## 5. Conclusions

Chronic heat stress significantly impacts the physiological and molecular homeostasis of *Gymnocypris eckloni*, a cold-adapted fish species highly vulnerable to climate warming. Our integrative multi-omics approach reveals that while gross growth parameters remain largely unaffected, heat stress induces notable histopathological damage in the liver and triggers a complex transcriptional and metabolic reprogramming. The suppression of core energy metabolism pathways, including amino acid, fatty acid, glucose, and oxidative phosphorylation metabolism, indicates a systemic downregulation of energy-intensive processes, likely as a survival strategy to conserve resources under thermal challenge. The integration of miRNA-mRNA-metabolite networks highlights conserved regulatory axes such as miR-196a-5p-*pepck*-phosphoenolpyruvate in gluconeogenesis and miR-132-3p-*ugt1a1*-bilirubin in detoxification, providing mechanistic insights into metabolic adaptation. Overall, this study demonstrates that *Gymnocypris eckloni* responds to thermal stress through a highly coordinated network involving miRNA-mediated gene regulation, metabolic pathway modulation, and cellular repair mechanisms. The identification of key pathways—particularly glutathione and tryptophan metabolism—provided valuable biomarkers for monitoring thermal stress in cold-water fish and highlights potential targets for assessing resilience in the face of climate change.

## Figures and Tables

**Table 1:** Functionally enriched pathways associated with miRNA-mRNA regulatory relationships.

miRNA	Gene	KEGG Pathway
miR-181b-5p	*nhp2*	Ribosome biogenesis in eukaryotes
miR-125b-2-3p	*ranbp2*	RNA transport
	*calr*	Phagosome, protein processing in endoplasmic reticulum
novel_582	*rpl34*	Ribosome
	*agl*	Starch and sucrose metabolism
miR-132-3p	*ugt1a1*	Starch and sucrose metabolism, porphyrin and chlorophyll metabolism, ascorbate and aldarate metabolism, pentose and glucuronate interconversions, retinol metabolism, steroid hormone biosynthesis, drug metabolism—cytochrome P450, metabolism of xenobiotics by cytochrome P450
miR-205-5p	*rgn*	Carbon metabolism, ascorbate and aldarate metabolism, pentose phosphate pathway
	*pdxp*	Vitamin B6 metabolism
	*adcy8*	Purine metabolism, progesterone-mediated oocyte maturation, GnRH signaling pathway, vascular smooth muscle contraction, gap junction, melanogenesis, oocyte meiosis
miR-196a-5p	*pepck*	Pyruvate metabolism, TCA cycle, PPAR signaling pathway, glycolysis/gluconeogenesis, adipocytokine signaling pathway, FoxO signaling pathway, insulin signaling pathway
miR-133a-3p	*bpnt1*	Sulfur metabolism
	*gne*	Amino sugar and nucleotide sugar metabolism

**Table 2:** Integrated analysis of metabolites and transcriptome.

Metabolic Pathway	*p* Value	Association with Transcriptome	Metabolites
Steroid hormone biosynthesis	0.002999	Yes	Cortisol, 5-beta-Androstane-3,17-dione, deoxycorticosterone, hydrocortisone, corticosterone, tetrahydrocortisone, tetrahydrocorticosterone
Tryptophan metabolism	0.01117	Yes	Indole, N-Formylkynurenine, L-Tryptophan, indole-3-acetic acid
Pyruvate metabolism	0.03314	Yes	acetyl phosphate, phosphoenolpyruvate
Aldosterone-regulated sodium reabsorption	0.03314	No	Hydrocortisone, cortisol
Phenylalanine, tyrosine, and tryptophan biosynthesis	0.03928	No	Phosphoenolpyruvate, L-Tyrosine, L-tryptophan, indole
cGMP-PKG signaling pathway	0.00276	No	Guanosine monophosphate, adenosine, adenosine 5′-monophosphate,
AMPK signaling pathway	0.00998	No	NAD^+^, adenosine 5′-monophosphate, D-Fructose 6-phosphate
cAMP signaling pathway	0.02040	No	adenosine 5′-monophosphate, adenosine
Longevity regulating pathway	0.02040	No	NAD^+^, adenosine 5′-monophosphate
Olfactory transduction	0.02040	No	Guanosine monophosphate, adenosine 5′-monophosphate
Purine metabolism	0.03753	Yes	XMP, adenosine 5′-monophosphate, adenine, adenosine, deoxyguanosine, guanosine monophosphate

## Data Availability

The original contributions presented in this study are included in the article/Supplementary Material. Further inquiries can be directed to the corresponding author.

## Supplementary Materials

The following supporting information can be downloaded at https://www.mdpi.com/article/10.3390/ani15223272/s1, Figure S1. Volcano plots showing differentially expressed genes (A) and miRNAs (B) identified between the two treatment groups. Table S1. Genes and specific primers used for qPCR. Table S2. Data output quality list of mRNAs. Table S3. Data output quality list of small RNA. Table S4. The DEMs of HTL vs. CTL. Table S5. miRNA-mRNA and the relative pathway.
