# Supplementary material for: Physiological Responses to Thermal Stress in the Liver of Gymnocypris eckloni Revealed by Multi-Omics
Source: Animals (Basel). 2025 Nov 12;15(22):3272. doi: 10.3390/ani15223272 (PMC12649434; doi:10.3390/ani15223272)
Supplement: Supplementary file 1 [file animals-15-03272-s001.zip › Supplementary figures.pdf]

Physiological responses to thermal stress in the liver of *Gymnocypris eckloni* revealed by multi-omics

Miaomiao Nie[1], Weilin Ni[1], Zhenji Wang[2], Dan Liu[1], Qiang Gao[1], Cunfang Zhang[1], Delin Qi[1*]

[1]State Key Laboratory of Plateau Ecology and Agriculture, Qinghai University, Xining 810016, China.

[2] Fishery Environmental Monitoring Station of Qinghai Province, Xining, 810012, China

* Correspondence: delinqi@126.com

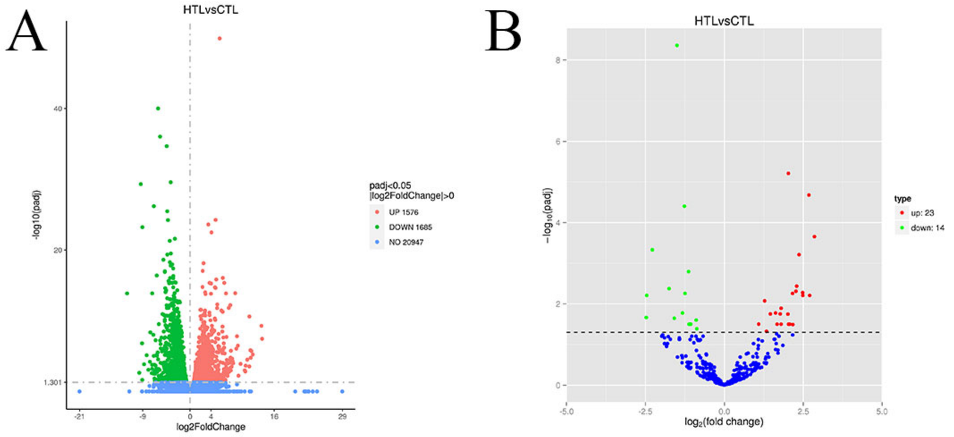

Figure S1. Volcano plots showing differentially expressed genes (A) and miRNAs (B) identified between the two treatment groups. Red and green circles indicate significantly upregulated and downregulated differences, respectively ($P < 0.05$), while blue circles indicate no differences.
