# Supplementary material for: Physiological Responses to Thermal Stress in the Liver of Gymnocypris eckloni Revealed by Multi-Omics
Source: Animals (Basel). 2025 Nov 12;15(22):3272. doi: 10.3390/ani15223272 (PMC12649434; doi:10.3390/ani15223272)
Supplement: Supplementary file 1 [file animals-15-03272-s001.zip › Supplementary tables.pdf]

Physiological responses to thermal stress in the liver of *Gymnocypris eckloni* revealed by multi-omics

Miaomiao Nie[1], Weilin Ni[1], Zhenji Wang[2], Dan Liu[1], Qiang Gao[1], Cunfang Zhang[1], Delin Qi[1*]

[1]State Key Laboratory of Plateau Ecology and Agriculture, Qinghai University, Xining 810016, China.

[2] Fishery Environmental Monitoring Station of Qinghai Province, Xining, 810012, China

* Correspondence: delinqi@126.com

Table S1 Genes and specific primers used for qPCR

Table S2 Data output quality list of mRNAs

Table S3 Data output quality list of small RNA

Table S4 The DEMs of HTL vs. CTL

Table S5 miRNA-mRNA and the relative pathway

Table S1 Genes and specific primers used for qPCR

Gene Name		Sequence (5'--3')
SPAM1	-F	AATTCCAGCAAGAAGCCAGA
	-R	TGCTTCCACTCACAGAAACG
GYS2	-F	CCTGCCAAAACCAACAACTT
	-R	TTGCATAAATGGCCCTCTTC
STS	-F	GGATTCGGATACCAGGGATT
	-R	TGTTCAGAACGCTCCACTTG
ApoB	-F	CGGAGACTCTGGACTTGAGG
	-R	TCCATGCCAATCTCAATGAA
SLC16A7	-F	CCCCAAATCCCTTACCATCT
	-R	AATGGTGGTGCCAAAAGAAG
HSP70	-F	CAACGGCAGAGACTTGAACA
	-R	AGGTCTGGGTCTGTTTGGTG
eIF4E	-F	AATAAGACGGCCAGTGACCA
	-R	GCCTCCAGTTGCATTCACAA
HSP40	-F	CTGGGGATAAAGAAAGGCGC
	-R	TGAAATTACCACCTCCGCCT
ATP1B	-F	ATGACGATGGAGTGCAGGAC
	-R	GGCAGATACCCGAGAATCCG
AMPKα1	-F	TCAAACTCTTCCGTCACCCG
	-R	GCCTGCTCTCCTTCTCATCC
AMPKα2	-F	CTCAACACCACTCGACCCAA
	-R	TTCTTCCGACGCACTCTCAG
AMPKβ1	-F	CAGACTTATCCAGCTCCCCA
	-R	CACATGGTTTGGTTCAGGCA
AMPKβ2	-F	TGTTTTCTCTCCAGCCGGTC
	-R	GCGTACGAACAGCGTCTCTA
AMPKγ1	-F	AATCTGGCCGCTGAGAAGAC
	-R	CCTCCTGCTCATCCACGATC
AMPKγ2	-F	CCCGATTCAGAAACAGCCCT
	-R	CCTGGCCCGAGAGAAAAGTT
ATP1B	-F	ATGACGATGGAGTGCAGGAC
	-R	GGCAGATACCCGAGAATCCG
PGC1α	-F	ATGCCAGAACAGGTGACAGG
	-R	CACTGGTGGGGCTTGATCAT
TFAM	-F	CGGGGGTCCACCTAAAAGAC
	-R	TGTTCAGGAGTCAGCGCTTT
PKA	-F	GGCACCAGGAGAAAGGACTA
	-R	GCCAGTGCCAAGAGTTTTCA
NRF1	-F	CACTCACAGCATTGCACACC
	-R	ATCTCGCCTCCCTGTAGTGT
β-actin	-F	GAACCCCAAGGCTAACAGAGAA
	-R	AGGCATACAGGGACAGCACA

Table S2 Data output quality list of mRNA

Sample	Raw Reads	Clean reads	Clean bases	Error (%)	Q20 (%)	Q30 (%)	Total map
HTL1	42219212	39329330	5.9G	0.02	98.88	96.17	31697866(80.6%)
HTL2	49045596	48315740	7.25G	0.02	98.34	95.01	38906303(80.53%)
HTL3	46496178	45185614	6.78G	0.02	98.86	96.06	37467148(82.92%)
CTL1	45204270	43433084	6.51G	0.02	98.78	96.01	33934113(78.13%)
CTL2	44660010	42804320	6.42G	0.02	98.81	96.08	32228330(75.29%)
CTL3	45921590	44146328	6.62G	0.02	98.66	95.58	33879021(76.74%)

Q20/Q30: the percentage of base, whose Phred values greater than 20 / 30; Q_{phred}=-10$\log_{10}$(e).

Table S3 Data output quality list of small RNA

Sample	Total reads	Clean reads	Bases (G)	Error rate (%)	Q20 (%)	Q30 (%)	GC (%)
HTL1	14165449 (100.00%)	13594738 (95.97%)	0.708G	0.01	99.47	97.84	49.40
HTL2	14368388 (100.00%)	13345165 (92.88%)	0.718G	0.01	99.46	97.90	49.98
HTL3	12940565 (100.00%)	12653894 (97.78%)	0.647G	0.01	99.47	97.87	49.34
CTL1	11322141 (100.00%)	10319076 (91.14%)	0.566	0.01	99.50	97.96	50.16
CTL2	12850052 (100.00%)	12490489 (97.20%)	0.643	0.01	99.50	97.97	50.34
CTL3	11578295 (100.00%)	10352581 (89.41%)	0.579	0.01	99.42	97.63	50.19
